# Uncovering the source of mitochondrial superoxide in pro-inflammatory macrophages: Insights from immunometabolism

**DOI:** 10.1016/j.bbadis.2022.166481

**Published:** 2022-07-02

**Authors:** Alva M. Casey, Michael P. Murphy

**Affiliations:** MRC Mitochondrial Biology Unit, Biomedical Campus, University of Cambridge, Cambridge CB2 0XY, UK

**Keywords:** Macrophages, Mitochondria, Superoxide, Metabolism, Complex I, Reverse electron transport

## Abstract

Mitochondrial-derived reactive oxygen species are important as antimicrobial agents and redox signals in pro-inflammatory macrophages. Macrophages produce superoxide in response to the TLR4 ligand LPS. However, the mechanism of LPS-induced superoxide generation is not fully understood. Superoxide is produced at complex I and complex III of the electron transport chain. Production of superoxide at either of these sites is highly dependent on the metabolic state of the cell which is dramatically altered by TLR4-induced metabolic reprogramming. This review will outline how metabolism impacts superoxide production in LPS-activated macrophages downstream of TLR4 signalling and address outstanding questions in this field.

## 1 Introduction

Macrophages are critical mediators of the innate immune system [1,2]. They are broadly classified as either classically activated (M1), pro-inflammatory macrophages or alternatively activated (M2), anti-inflammatory macrophages, although this distinction has recently become less discrete. Pro-inflammatory macrophages are activated in response to pathogen associated molecular patterns (PAMPs), such as lipopolysaccharide (LPS), or danger associated molecular patterns (DAMPs), and play an important role in the immediate response to infection [3].

Toll-like receptor 4 (TLR4)-activated macrophages adopt a specific metabolic signature characterised by suppression of oxidative phosphorylation (OXPHOS), an upregulation of glycolysis and an accumulation of the TCA cycle metabolites itaconate and succinate [4–6]. LPS-stimulated macrophages also produce enhanced levels of superoxide and hydrogen peroxide. These oxidants are important to macrophage immunity, both as bactericidal agents and as redox signals. It was previously thought that the primary source of reactive oxygen species (ROS) in activated macrophages was NADPH oxidase 2 [7–9]. However, mitochondria are also an important source of superoxide and hydrogen peroxide downstream of TLR4 signalling [10]. In this review, we use the terms mitochondrial ROS (mtROS) or ROS to refer to hydrogen peroxide and superoxide [11].

The major production sites of superoxide within mitochondria are complex I and complex III of the electron transport chain (ETC) [12].

However, the relative contribution of these sites to superoxide generation in TLR4-activated macrophages is unclear. It is well established that mitochondrial properties and cellular metabolism significantly impact both the location and extent of mitochondrial superoxide production [12,13]. Therefore, we will outline the influence of the metabolic phenotype of TLR4-activated macrophages on mitochondrial superoxide generation and highlight how the study of immunometabolism can help delineate the source and role of mitochondrial superoxide in pro-inflammatory macrophages.

## 2 Complex I as a source of mtROS

Complex I of the mitochondrial ETC is an important source of superoxide. Several studies have implicated complex I as being critical for macrophage function and shown that complex I dysfunction disrupts mtROS production in pro-inflammatory macrophages [10,14–16]. Inhibition of complex I with either metformin or rotenone decreases LPS-induced mtROS production [15]. This points to complex I involvement in superoxide production downstream of TLR4 signalling. Furthermore, TLR4 activation also enhances complex I assembly [10]. TLR1/2/4 agonists cause tumor necrosis factor receptor-associated factor 6 (TRAF6) to translocate to the mitochondria where it interacts with evolutionarily conserved signalling intermediate in Toll pathways (ECSIT). ECSIT interacts with the complex I assembly factors ACAD9 (Acyl CoA dehydrogenase family member 9) and NDUFAF1 (NADH dehydrogenase [ubiquinone] 1 alpha subcomplex assembly factor 1) suggesting that ECSIT forms part of the mitochondrial complex I assembly (MCIA) complex in macrophages [14]. Conditional *Ecsit*-knockout bone marrow derived macrophages (BMDMs) have an increased baseline mtROS but do not demonstrate an LPS-induced increase in mtROS production [14]. Moreover, *Traf6*- and *Ecsit*-knockdown BMDMs have a reduced bactericidal capacity when exposed to Salmonella typhimurium. Since NADPH-oxidase activity, nitric oxide production and pro-inflammatory cytokine levels were comparable in controls and knockdown BMDMs, this demonstrates that complex I activity is needed for macrophage effector functions [10]. Therefore, a detailed understanding of how superoxide is generated at complex I and the factors that influence its production is crucial if we are to resolve the involvement of complex I-derived superoxide in TLR4-activated macrophages.

## 3 Mechanism of superoxide production at complex I

**Fig. 1:**
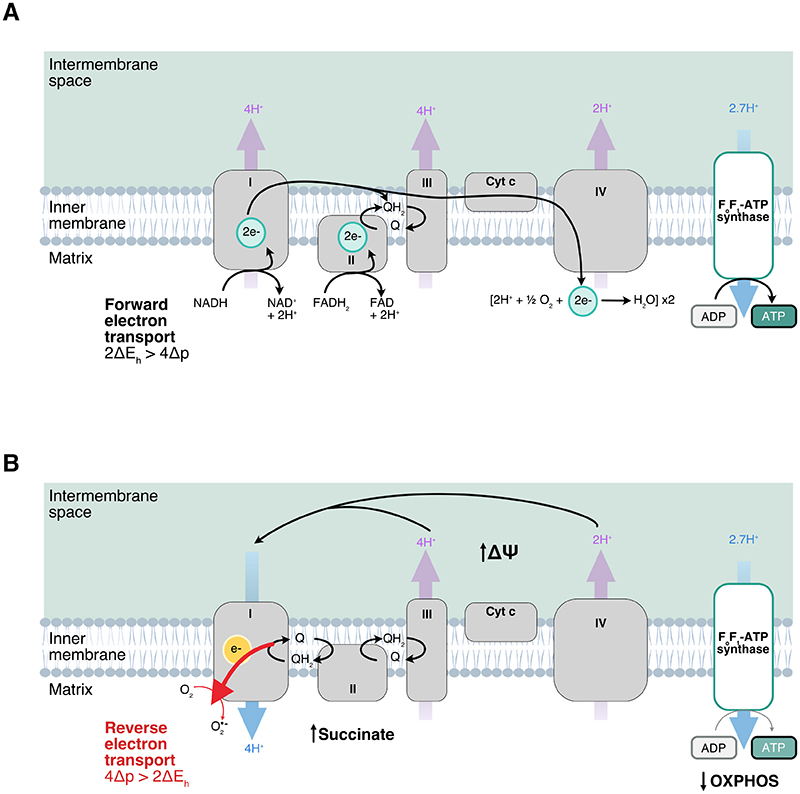
Superoxide production by reverse electron transport at complex I. A) Forward electron transport at complex I. Complex I accepts two electrons from NADH which then reduce CoQ to CoQH_2_. The energy derived from the transfer of electrons along the electron transport chain from complexes of increasing reduction potential drives proton pumping across the mitochondrial inner membrane to generate the proton motive force (Δp) that is used to produce ATP by oxidative phosphorylation. Forward electron transfer at complex I occurs when the difference in reduction potential between the NAD^+^/NADH and CoQ/CoQH_2_ couples (ΔE_h_) is greater than the energy required to pump protons against Δp. Since 4 protons (4H^+^) are pumped per pair of electrons (2e-) transported, the thermodynamic requirement for forward electron transfer is 2ΔE_h_ > 4Δp. B) Reverse electron transfer at complex I. Electrons are transferred from the CoQ pool to FMN on complex I and then to oxygen to generate superoxide (${\text{O}}_{2}^{●-}$) when 4Δp > 2ΔE_h_. These conditions are satisfied when there is a high potential (Δψ) across the mitochondrial inner membrane facilitated by decreased oxidative phosphorylation, accumulation of succinate and a reduced CoQ pool.

The mechanism of superoxide production upon LPS stimulation is not fully understood. One proposed mechanism of superoxide production at complex I in LPS-activated macrophages is via reverse electron transport (RET) [16]. The normal role of complex I is to accept electrons from NADH while pumping protons across the mitochondrial inner membrane to generate the proton motive force (Δp) which drives ATP synthesis (Fig. 1A). Forward electron transfer from complex I to the CoQ pool will only occur if the difference in reduction potential between the NAD^+^/NADH and the CoQ/CoQH_2_ couples (ΔE_h_) is sufficient to pump protons against Δp. Four protons are pumped per pair of electrons that are transferred through complex I. Therefore, the thermodynamic requirement for forward electron transfer is 2ΔE_h_ > 4Δp. A corollary is that electrons can be transferred in the reverse direction, from the CoQ pool through complex I to the flavin mononucleotide (FMN) when 4Δp > 2ΔE_h_. Electrons are transferred in the reverse direction from CoQ through complex I onto the FMN and are then passed to oxygen to produce superoxide (Fig. 1B) [12]. The conditions for 4Δp > 2ΔEh to be satisfied and RET to occur have been outlined by Chouchani et al. [17] in the context of ischemia/reperfusion injury. They include a high mitochondrial Δp, accumulation of succinate leading to succinate oxidation which maintains a reduced CoQ pool, and limited ATP synthesis by OXPHOS that would otherwise deplete the Δp [17]. These factors strongly promote superoxide production by RET at complex I. Not only that, but several seminal papers have outlined that TLR4 signalling results in changes in mitochondrial metabolism that give rise to such conditions in LPS-activated macrophages [16,18,19].

## 4 Factors that influence superoxide production by RET at complex I

Succinate accumulation and subsequent oxidation is associated with RET and ROS production at complex I [20]. Succinate accumulation is well characterised in LPS-stimulated macrophages and is primarily derived from a combination of glutamine anaplerosis via glutamate [18] and competitive inhibition of succinate dehydrogenase (SDH), also known as complex II, by itaconate [21,22]. Mills et al. [16] built on this finding and demonstrated that pre-treatment with the succinate dehydrogenase inhibitor dimethyl malonate (DMM) decreases mtROS production, indicating that succinate oxidation promotes mtROS production in LPS-activated macrophages.

A high Δp also promotes mitochondrial superoxide production and it has been shown that LPS activation leads to an increased mitochondrial membrane potential (the major component of the Δp) [16,19]. Decreased ATP synthesis by OXPHOS favours mtROS production because Δp will remain high when protons do not flow across the mitochondrial inner membrane via the F_o_F_1_-ATP synthase [17]. Metabolic reprogramming in LPS-activated macrophages that results in a shift in ATP production from OXPHOS to glycolysis will enhance this effect. The presence of a high Δp (usually measured as a high mitochondrial membrane potential) in LPS-activated macrophages is an important requirement for superoxide production by RET at complex I.

**Fig. 2:**
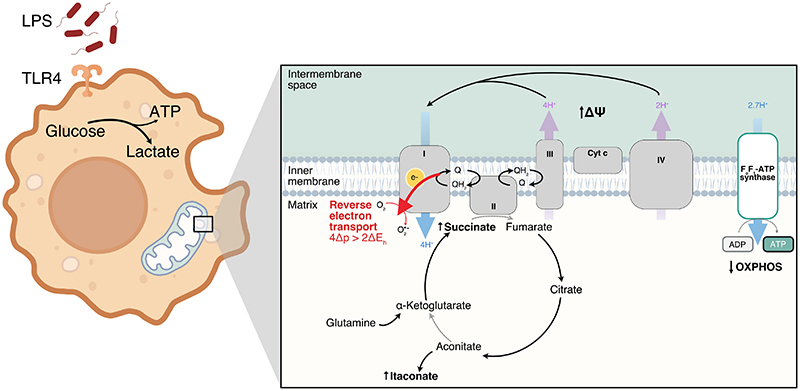
The influence of LPS induced metabolic reprogramming on the production of mitochondrial superoxide. Lipopolysaccharide (LPS) binds to its cognate receptor TLR4. TLR4 signalling leads to upregulation of glycolysis, a reduction in ATP production by oxidative phosphorylation (OXPHOS) and accumulations of succinate via glutaminolysis and itaconate produced via aconitate. There is also an increase in potential (Δψ) across the mitochondrial inner membrane. Accumulation of succinate and a high membrane potential drives reverse electron transfer and superoxide (${\text{O}}_{2}^{●-}$) production at complex I of the electron transport chain.

Together, succinate accumulation, reduced F_o_F_1_-ATP synthase activity and a high Δp have been shown to enhance mtROS production by RET at complex I in ischemia/reperfusion injury [17]. These conditions are generated downstream of TLR4 signalling by enhanced succinate generation and suppression of mitochondrial F_o_F_1_-ATP synthase activity by the activation of glycolysis (Fig. 2). Therefore, complex I is a strong candidate source of superoxide production in LPS-activated macrophages and these data support the model of superoxide production by RET.

## 5 Complex III as an alternative source of superoxide

Complex III of the mitochondrial ETC is also a source of superoxide and has been suggested to influence pro-inflammatory macrophage effector functions. At complex III, electrons are transferred from the CoQ pool to cytochrome *c* via the Q-cycle. Generation of superoxide has been demonstrated at the Q_o_ site of complex III by the reaction of oxygen with ubisemiquinone at the Q_o_ site to produce superoxide. However, substantial superoxide production at Qo has only been demonstrated when complex III is inhibited by Q_i_ inhibitors such as antimycin A. There are several factors that could influence superoxide generation at complex III in the absence of Q_i_ inhibitors. A reduced CoQ pool and a high Δp also promotes superoxide production because it slows down transfer of an electron from heme bl to heme b_h_ [23]. This enhances the lifetime of the ubisemiquinone at the Q_o_ site leading to enhanced superoxide production at the Q_o_ site. However, both a high Δp and a reduced CoQ pool will also promote RET at complex I, therefore drawing electrons away from complex III. Hence, the physiological relevance of superoxide production at complex III is thought to be lower than that of complex I [12] although some studies have suggested that complex III-derived superoxide influences LPS-activated macrophage functions [24,25].

Treatment with myxothiazol, a Q_o_ site inhibitor, reduces mtROS production in LPS/IFN-γ-stimulated macrophages. Myxothiazol results in changes to the activation of LPS/IFN-γ-treated macrophages including reduction in the expression of MHC class II, CD80 and NOS2, as well as the expression of the pro-inflammatory cytokines pro-IL-1β, IL-6 and TNF [25]. This indicates that complex III activity could play a role in macrophage effector functions, and as is discussed by Billingham and Chandel [24], it is in contrast to the work of Mills et al. [16] that demonstrated that IL-1β induction was dependent on complex I-derived superoxide. However, myxathiozol treatment could also affect superoxide production by RET at complex I. Inhibition of electron transfer from ubiquinol to cytochrome *b* prevents proton pumping at both complex III and complex IV, thus lowering Δp and negatively impacting RET. Since RET is dependent on 4Δp > 2ΔE_h_, the study of complex III-derived superoxide using inhibitors of complex III that result in a reduction in Δp will also impact superoxide production by RET at complex I.

Care must also be given to experimental design when studying RET at complex I. Mills et al. [16] used BMDMs from mice expressing the alternative oxidase (AOX) which oxidizes the CoQ pool thereby preventing RET, to argue that a reduced CoQ pool contributes to RET-derived superoxide at complex I, based on the knowledge that RET is dependent on a reduced CoQ pool (4Δp > 2ΔEh). However, Billingham and Chandel [24] point out that AOX would also affect the generation of superoxide at complex III. Similarly, using rotenone to block complex I will block electrons entering the CoQ pool from complex I and therefore affect superoxide production at complex III [15].

To further complicate this area, studies on ECSIT have called into question the role of complex III in LPS-induced superoxide generation. ECSIT conditional knock out BMDMs had higher baseline ROS levels which is consistent with complex I dysfunction [14]. The conditional knockout macrophages also retained the ability to produce complex III-derived mtROS, as evident by an increase in ROS upon antimycin A treatment. However, there was no further increase in ROS upon LPS stimulation of ECSIT-deleted macrophages suggesting that complex III does not contribute to LPS-induced mtROS production. While IL-1β expression was not examined, neither TNF-α nor IL-6 expression changed [14].

As is evident from the contradictory interpretations of the studies described, it can be difficult to distinguish between complex I and complex III as the sites of superoxide production because interference using inhibitors or experimental conditions that inhibit complex III superoxide production often also impact RET at complex I. The use of genetic models that are unable to produce superoxide by RET, such as is the case with the *ND6* G14600A mtDNA mutation that leads to a proline to leucine substitution at position 25 in the ND6 subunit of complex I (ND6-P25L), will be able to provide a clearer picture of the mechanism of generation of LPS-induced superoxide [26–28].

It is also important to consider the time frame of the experimental design of these studies when interpreting the results. As we have outlined above, changes in metabolism and altered mitochondrial function significantly impact superoxide production. What we have not yet emphasised is that LPS activation results in highly dynamic changes in macrophage metabolism and mitochondrial properties. Therefore, it is critical to consider the length of exposure to LPS and the corresponding metabolic state of the TLR4-activated macrophage when interpreting these results.

## 6 The effect of dynamic metabolic reprogramming on mtROS production

It is clear that the metabolic state of LPS-activated macrophages has a significant impact on mitochondrial superoxide production, and proinflammatory macrophages undergo highly dynamic metabolic reprogramming. Seim et al. [29] describe the metabolome of LPS/IFN-γ-activated macrophages over 72 h to illustrate the dynamic nature of metabolic reprogramming in activated RAW 264.7 cells and murine BMDMs. They characterise two distinct metabolic stages following LPS/IFN-γ-stimulation which they call early stage (6-24 h treatment) and late stage (24-72 h treatment). They show that early LPS/IFN-γ treatment results in the characteristic ‘breaks’ in the TCA cycle with accumulations of itaconate, citrate and succinate while TNF-α and IL-6 expression is maximal. Continued exposure results in diminished TNF-α and IL-6 production and normalisation of citrate, itaconate and succinate levels. Acetyl-CoA and succinyl-CoA levels are also decreased at late stage LPS treatment [29]. Similarly to the alterations in mitochondrial metabolite abundance, LPS induced changes in mitochondrial membrane potential are also time dependent, with a slight increase in potential seen after only 30 mins LPS stimulation while maximal potential is thought to be achieved at 24 h LPS exposure [16].

Due to these dynamic changes, we draw particular attention to the time frames used in the above studies. Mills et al. [16] used a 3 h pretreatment with dimethyl succinate (DMS) or DMM followed by 48 h LPS stimulation to study complex I activity. In contrast, Cameron et al. [25] used 1 h pre-treatment of myxothiazol and then 1 h co-treatment with LPS/IFN-γ followed by a further 17 h LPS/IFN-γ stimulation in the absence of myxothiazol. West et al. [10], who demonstrated that TLR signalling induced an increase in mtROS production, stimulated macrophages with LPS over 8 h while Carneiro et al. [14] used just 20 min LPS stimulation. The conflicting results of these studies highlight the importance of considering the impact of progression of the metabolic phenotype on mitochondrial superoxide production in macrophages. This will also be critical when investigating superoxide production in response to secondary exposure to PAMPs in the context of immune memory [30].

Another caveat of these studies is that many of them use pretreatment with inhibitors that alter cellular metabolism that could lead to metabolic rewiring prior to LPS-induced metabolic reprogramming. This type of experimental design has different implications and mechanisms than being able to block superoxide production downstream of TLR4 signalling. An additional example of this type of experimental design is seen in a recent paper which investigates the role of mitochondria in NLRP3 inflammasome activation. This paper argues that ETC-derived ROS are not required for NLRP3 inflammasome activation but rather ETC inhibitors reduce phosphocreatine (PCr)-dependent generation of ATP which is necessary for inflammasome activation [31]. Activation of NLRP3 inflammasome in macrophages requires exposure to two stimuli: initial priming by LPS, followed by activation with stimuli that are either dependent or independent on potassium efflux [32,33]. In this study, BMDMs were treated with ETC inhibitors 30 mins prior to LPS priming [31]. Since LPS induces metabolic rewiring, pre-treatment with ETC inhibitors may result in a different metabolic phenotype and outcome compared to ETC inhibition after LPS priming and prior to inflammasome activation.

These studies demonstrate that our understanding of the dynamic nature and influence of mitochondrial metabolism and mitochondrial properties on superoxide production is fundamental to the study of superoxide production in pro-inflammatory macrophages. An outline of the time dependent metabolic changes in parallel with a study of LPS-induced superoxide production in macrophages is a fundamental gap which must be filled in this field.

## 7 The influence of mtROS and mitochondrial metabolites on macrophage effector functions

Elucidating how mitochondrial superoxide generation is influenced by metabolism, will no doubt provide insights into the role of mitochondrial derived superoxide in macrophage effector functions. Before it was appreciated that mitochondria produced ROS in pro-inflammatory macrophages it was evident that macrophages used reactive species as antibacterial agents [34]. We now know that mtROS are also used in a similar capacity because a reduction in LPS-induced mtROS is associated with decreased bactericidal ability of macrophages [10]. Similarly, prevention of the succinate oxidation that promotes mtROS production, by inhibiting SDH, promotes an anti-inflammatory macrophage phenotype in models of sepsis [16] and Escherichia coli infection [35]. Furthermore, TLR4-induced mtROS production can be directly targeted to phagosomes to be used as an antimicrobial agent [10]. Therefore, the study of the mechanism of mtROS generation in LPS-activated macrophages will provide broader insights into how macrophages fight infection.

Here, we have highlighted how metabolism influences the production of LPS-induced mtROS. However, as well as driving mtROS production, metabolites can also act as effector molecules in innate immunity. Therefore, it will be interesting to study mtROS production in the context of immunometabolite signalling. A clear example of the interplay between mtROS and metabolite signalling is found in succinate dependent promotion of IL-1β production in pro-inflammatory macrophages. Succinate stabilises HIF-1α, which promotes IL-1β production, both by directly inhibiting prolyl hydroxylase (PHD) and by promoting mtROS production by RET which also inhibit PHD activity [16,18]. Succinate then promotes the sustained production of IL-1β, independently of mtROS signalling, by acting as an autocrine and paracrine molecule. Mice deficient in the G-protein coupled receptor, succinate receptor 1 (SUCNR1; previously known as GPR91) demonstrated impaired IL-1β production with either LPS activation or in a model of rheumatoid arthritis [36]. Therefore, not only will the study of immunometabolism help us to uncover the mechanism of LPS-induced mtROS production but it will also allow us to place mtROS production in the context of metabolite signalling in pro-inflammatory macrophages.

Finally, we would also like to highlight that generation of mtROS in pro-inflammatory macrophages is associated with many pathologies and biomedically important processes. For example, mtROS are important in the early stages of wound healing for pro-angiogenic wound macrophage function [19] while increased mtROS production is associated with increased inflammation and abrogated osteocyte differentiation and bone resorption in bone degenerative diseases [37]. Treatment of LPS-activated macrophages with the complex I inhibitor metformin reduces mtROS production [15] and may be associated with its efficacy as a widely used drug to treat type II diabetes mellitus. Furthermore, since mtROS is associated with production of the pro-inflammatory cytokine IL-1β in many inflammatory disorders, drugs such as metformin may be used in the future as anti-inflammatory therapeutics [38]. Understanding the factors that control the production and origin of mtROS will allow us to develop targeted therapeutics.

## 8 Conclusion and future directions

Here we have outlined that the metabolic state of macrophages strongly influences mtROS production and put forward suggestions about how this can lead us to define the source of mtROS production in TLR4-stimulated macrophages. We have described how the conditions induced by TLR4 signalling promote superoxide production by RET at complex I [16]. In future years, it will be important to test the validity of this model and to clarify the role, if any, that complex III plays in mtROS production in LPS-activated macrophages.

We highlight that our understanding of the dynamic nature of metabolic reprogramming is important so outlining a clear model and time course of metabolic change in the context of mtROS is critical. Another consideration is that many studies investigating mtROS use non-specific probes for cellular ROS as a proxy measurement. It is crucial to directly measure mtROS and to define the particular species being studied [11]. We also draw attention to measurements of mitochondrial membrane potential using dyes, the uptake of which is dependent not only on the mitochondrial membrane potential but also on the plasma membrane potential. Measurements of the plasma membrane potential are an overlooked control that is necessary for proper interpretation of these results [39,40]. These experimental considerations will be critical if we are to unveil the mechanism of superoxide production in LPS-activated macrophages and to understand the role of mtROS in the context of innate immunity and inflammatory disease.

## Abbreviations

ACAD9: Acyl-CoA dehydrogenase family member 9
AOX: alternative oxidase
ATP: adenosine triphosphate
BMDM: bone marrow derived macrophage
CD80: cluster of differentiation 80
CoQ: Coenzyme Q
DAMP: danger associated molecular pattern
ΔE_h_: reduction potential
Δp: proton motive force
DMM: dimethyl malonate
DMS: Dimethyl succinate
ECSIT: evolutionarily conserved signalling intermediate in Toll pathway
ETC: electron transport chain
FMN: Flavin mononucleotide
HIF-1α: Hypoxia inducible factor 1 subunit alpha
IFN-γ: interferon-γ
IL-1β: interleukin-1β
IL-6: interleukin-6
LPS: lipopolysaccharide
MCIA: complex mitochondrial complex I assembly complex
MHC: major histocompatibility complex
mtROS: mitochondrial reactive oxygen species
NADH: dihydronicotinamide adenine dinucleotide
NADPH: nicotinamide adenine dinucleotide phosphate
ND6: NADH dehydrogenase 6
NDUFAF1: (NADH dehydrogenase [ubiquinone] 1 alpha subcomplex assembly factor 1)
NLRP3: NOD-, LRR- and pyrin domain-containing protein 3
NOS2: nitric oxide synthase 2
OXPHOS: oxidative phosphorylation
PAMP: pathogen associated molecular pattern
PCr: Phosphocreatine
PDH: prolyl hydroxylase
RET: reverse electron transport
ROS: reactive oxygen species
SCNR1: succinate repector 1
SDH: succinate dehydrogenase
TCA: tricarboxcylic acid cycle
TLR: Toll-like receptor
TNF: tumor necrosis factor
TRAF: tumor necrosis factor receptor-associated factor

## Data Availability

Data will be made available on request.
